# Recommendations for clinical research in children presenting to primary care out-of-hours services: a randomised controlled trial with parallel cohort study

**DOI:** 10.3399/bjgpopen20X101154

**Published:** 2021-02-17

**Authors:** Anouk AH Weghorst, Gea A Holtman, Pien I Wolters, Heleen A Russchen, Freek Fickweiler, Henkjan J Verkade, Johan Post, Karin M Vermeulen, Boudewijn J Kollen, Irma J Bonvanie, Marjolein Y Berger

**Affiliations:** 1 Department of General Practice and Elderly Care Medicine, University of Groningen, University Medical Centre Groningen, Groningen, The Netherlands; 2 Department of Paediatrics, University of Groningen, University Medical Centre Groningen, Groningen, The Netherlands; 3 Department of Out-Of-Hours Service Groningen, Groningen, The Netherlands; 4 Department of Epidemiology, University of Groningen, University Medical Centre Groningen, Groningen, The Netherlands

**Keywords:** Primary care, Informed consent, Off-protocol, Selection bias, Children, Randomised controlled trial, Cohort

## Abstract

**Background:**

Research in primary care is essential, but recruiting children in this setting can be complex and may cause selection bias. Challenges surrounding informed consent, particularly in an acute clinical setting, can undermine feasibility. The off-protocol use of an intervention nearing implementation has become common in pragmatic randomised controlled trials (RCTs) set in primary care.

**Aim:**

To describe how the informed consent procedure affects study inclusion and to assess how off-protocol medication prescribing affects participant selection in a paediatric RCT.

**Design & setting:**

A pragmatic RCT evaluating the cost-effectiveness of oral ondansetron in children diagnosed with acute gastroenteritis (AGE) in primary care out-of-hours services and a parallel cohort study.

**Method:**

Consecutive children aged 6 months to 6 years attending primary care out-of-hours services with AGE were evaluated to assess the feasibility of obtaining informed consent, the off-protocol use of ondansetron, and other inclusion and exclusion criteria.

**Results:**

The RCT's feasibility was reduced by the informed consent procedure because 39.0% (*n* = 325/834) of children were accompanied by only one parent. GPs prescribed ondansetron off-protocol to 34 children (4.1%) of which 19 children were eligible for the RCT. RCT-eligible children included in the parallel cohort study had fewer risk factors for dehydration than children in the RCT despite similar dehydration assessments by GPs.

**Conclusion:**

The informed consent procedure and off-protocol use of study medication affect the inclusion rate, but had little effect on selection. A parallel cohort study alongside the RCT can help evaluate selection bias, and a pilot study can reveal potential barriers to inclusion.

## How this fits in

Research in primary care is essential for evidence-based health care, yet 40% of paediatric RCTs are discontinued prematurely. A pilot study can help to identify potential recruitment barriers and prevent many reasons for recruitment failure. However, based on this study, the authors further advocate: 1) reconsidering the need for written informed consent from both parents in trials where the risk to the child is low; and 2) improving communication with all stakeholders to prevent off-protocol prescribing of study medication. Finally, selection bias can be identified by using a parallel cohort study.

## Introduction

Outcomes from RCTs are essential for GPs to provide evidence-based health care.^1^ However, recruiting sufficient numbers of representative participants can be difficult, especially for acute paediatric management.^2^ This is illustrated by the fact that 40% of paediatric RCTs are discontinued prematurely owing to poor recruitment.^2–4^ Besides that, GPs who are aware of the effectiveness of the intervention may use this intervention, which can cause selection bias.^5^

Primary care is not an easy place to conduct research.^6^ Although GP involvement in case recruitment can decrease the chance of successful inclusion,^1^ not involving them is not always feasible and can be costly. In out-of-hours primary care centre (OOH-PC), GPs must also evaluate patients with whom they are unfamiliar, which may further decrease their willingness to recruit children into a trial and worsen the inclusion rate.^1^ When trials are discontinued, authors rarely report how and by whom participants were recruited, which prevents any lessons learnt being applied when planning trials in other settings.^2^ Ideally, authors would report the recruitment process of their trial in sufficient detail to help avoid the repetition of mistakes.^5^ Pilot studies can be used to uncover reasons for recruitment failure.^5^

A pragmatic RCT was performed to investigate the cost-effectiveness of adding a single dose of oral ondansetron to care-as-usual (CAU) in an OOH-PC on the frequency of vomiting in children aged 6 months to 6 years with AGE. Despite a pilot study, child recruitment was challenging. In this report, the recruitment efforts are described, focusing on how the informed consent procedure and the use of off-protocol prescribing affected the inclusion rate and child selection, respectively.

## Method

### Study design

In the pragmatic RCT, participants were enrolled from December 2015 to January 2018 at three OOH-PCs in the north of the Netherlands (Groningen, Zwolle, and Assen). After a pilot study (NL4700) from December 2015 to October 2016, in agreement with the medical ethical committee (METc), the primary outcome was changed from ‘referral rate’ to ‘proportion of children who continued vomiting in the first 4 hours after randomisation’ because this was considered a more patient-oriented outcome by both the METc and the parents involved. The primary and secondary outcomes of the RCT are detailed in Supplementary Box 1. The written informed consent procedure was also adapted because it could not be feasibly obtained from both parents, severely restricting inclusion. The METc agreed that children could be included from the pilot study in the amended RCT (NL5830), and a parallel cohort study was added. The parallel cohort study provides insight into the representativeness of the trial population and helps to assess the external validity in a non-invasive manner.^7,8^ Follow-up was for 7 days after randomisation.

### Participants

#### Inclusion criteria: RCT

Children aged 6 months to 6 years were included who presented at the OOH-PC with vomiting and for whom the GP diagnosed AGE. Specifically, children were included if: 1) they reported at least four episodes of vomiting 24 hours before presentation; 2) they reported at least one episode of vomiting 4 hours before presentation; and 3) written informed consent was obtained from both parents. If the child was accompanied by one parent, that parent could give written informed consent and the second parent could give oral informed consent via a telephone call in the presence of the research assistant (RA). The written informed consent from the second parent had to be sent by post.

#### Exclusion criteria: RCT

Children were excluded if: 1) they had used or been prescribed antiemetics in the previous 6 hours; 2) they had renal failure or hypoalbuminaemia; 3) they had diabetes mellitus or inflammatory bowel disease; 4) they had a history of abdominal surgery that could explain the current symptoms according to the GP; 5) they had sensitivity to 5-HT3 receptor antagonists; 6) they had a prolonged QT interval or were using QT-prolonging medication; or 7) they had previously been enrolled.

#### Inclusion criteria: parallel cohort study

Children aged 6 months to 6 years and diagnosed with AGE, but whose parents did not give written informed consent for the RCT, were asked if they were willing to participate in a parallel cohort study in which written informed consent was only needed from one parent.

### Patient recruitment and baseline assessment

**Figure 1.**
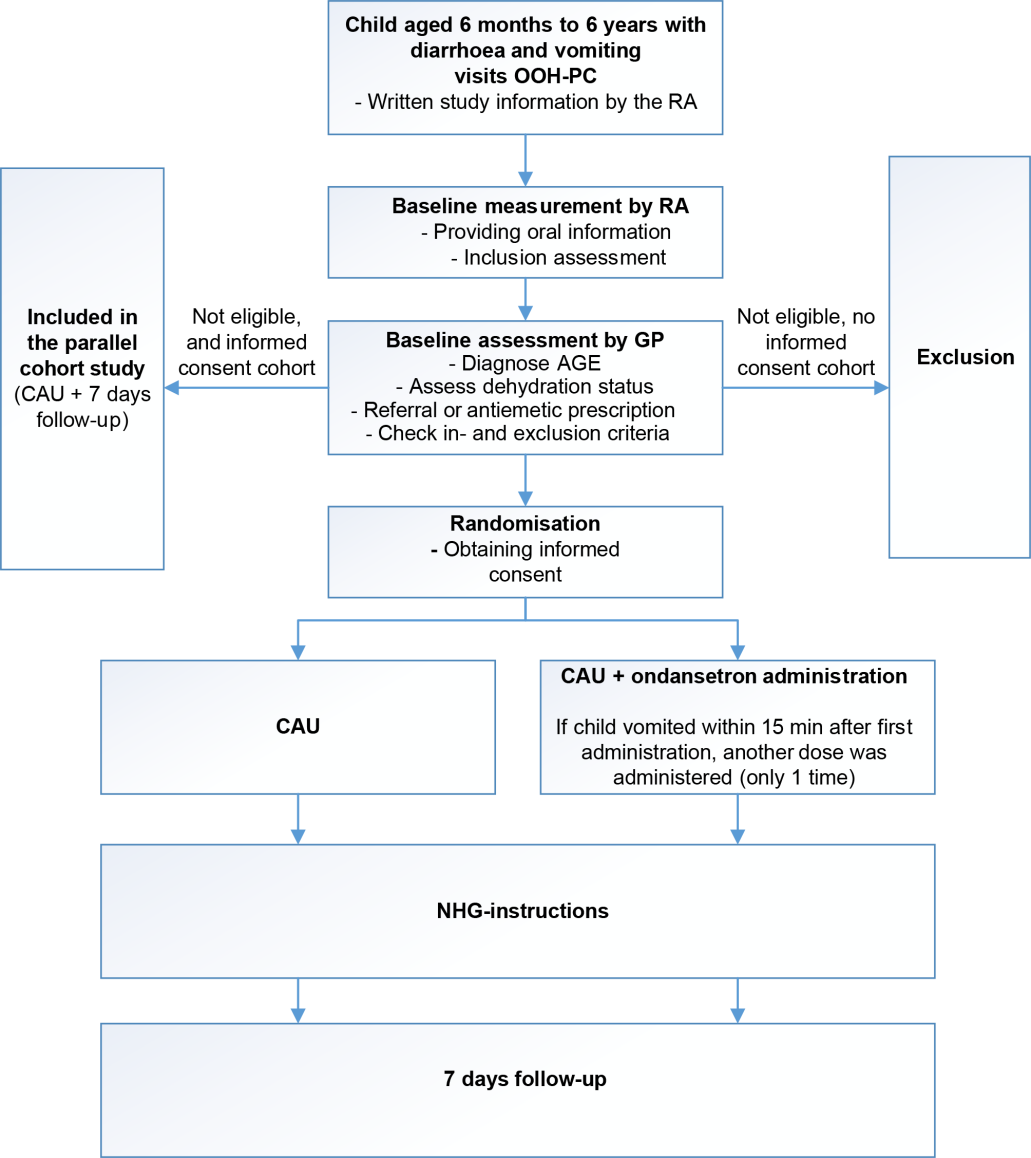
Study design. AGE = acute gastroenteritis. CAU = care as usual. OOH-PC = out-of-hours primary care. RA = research assistant.

Parents were informed about the study by an RA before the GP consultation. If parents were interested, the RA started baseline assessments (Supplementary Box 2) and the GP then assessed the child (confirming or refuting a diagnosis of AGE and assessing the degree of dehydration) and their suitability for participating based on the inclusion and exclusion criteria. The RA asked the parents of eligible children for written informed consent (Figure 1).

### Randomisation and blinding

After obtaining at least one written and one oral informed consent from parents, children were block randomised (1:1 allocation) to intervention groups by a computer programme and were stratified by age (6–24 months or >24 months) and dehydration status (see Table 1: ‘at risk’ if no alarm symptoms, or ‘dehydrated’ if ≥1 alarm symptom). Allocation was not generated before inclusion to ensure concealment. Treatment allocation was not blinded to the parents, the child, the GP, or the RA. The researcher who performed the statistical analyses was blinded to treatment allocation.

**Table 1.** Alarm symptoms and risk factors for dehydration

**Alarm symptoms of dehydration**	**Risk factors for dehydration**
Confused or decreased consciousness	≥6 watery stools or diarrhoea
Bradycardia	Fever (>38°C)
Weak peripheral pulse	Reduced intake in the last 12 hours
Capillary refill >4 seconds	
Skin pitch >4 seconds	
Extremities cold or marbled	
Reduced urine output in the last 24 hours	

### Interventions

#### Control group and parallel cohort study: CAU

Children received CAU (Figure 1) that comprised instructions to buy oral rehydration solution and how to use it, as described in the acute diarrhoea guideline of the Dutch College of General Practitioners.^9^ That involved 10 ml/kg compensation when at risk of dehydration (that is, all children) and 15 ml/kg for 4 hours when assessed as dehydrated. Parents were informed about the expected course, alarm symptoms, and when and how to contact their GP.

#### Intervention group: ondansetron plus CAU

Children in the intervention group received CAU plus a single weight-based dose of oral ondansetron syrup (0.1 mg/kg) according to the Dutch Paediatric Formulary.^10^ If the child vomited within 15 minutes, the dose was given a second time only.

### Follow-up assessment

Parents used a structured diary to record symptoms (that is, diarrhoea, vomiting, and fever), oral rehydration therapy and fluid intake, medication use, adverse reactions, healthcare use, hours missed from work, and satisfaction with treatment during follow-up. The diary was to be completed every hour for the first 4 hours and daily thereafter for 7 days. Parents could return the diary on paper with the enclosed envelope. If parents did not return the diary after multiple requests, information was collected about the primary outcome by telephone.

### Sample size

Based on a systematic review, it was estimated that 85% and 64% of children in the CAU and ondansetron groups would continue vomiting after 4 hours,^11^ indicating that a difference of 21% in the proportion of children with persistent vomiting was clinically relevant. Therefore, 89 children per arm needed to be included for a power of 90% and an α of 0.05, allowing for a 10% loss to follow-up.

### Statistical analysis

The following baseline characteristics were assessed: age, sex, AGE symptoms (weight, duration or frequency of vomiting and/or diarrhoea), dehydration alarm symptoms, dehydration risk factors (see Table 1),^9^ and degree of dehydration assessed by the GP (0–100 scale). To evaluate the impact of the informed consent procedure on the inclusion rate, the number of children with AGE who visited the OOH-PC with only one parent was reported. To evaluate the impact of the off-protocol prescription of ondansetron on the selection of children in the RCT, baseline characteristics of RCT-eligible children included in the parallel cohort study and for whom the GP prescribed ondansetron were non-statistically compared with children included in the RCT. To understand the overall level of selection bias, the baseline characteristics of RCT-eligible children included in the parallel cohort study were statistically compared with the baseline characteristics of children included in the RCT. Continuous variables were compared with non-parametric tests (Mann–Whitney U test) and dichotomous variables were compared by logistic regression. Statistical significance was defined by a two-tailed *P* value of <0.05, and all analyses were performed using SPSS (version 25).

## Results

### Study population

**Figure 2.**
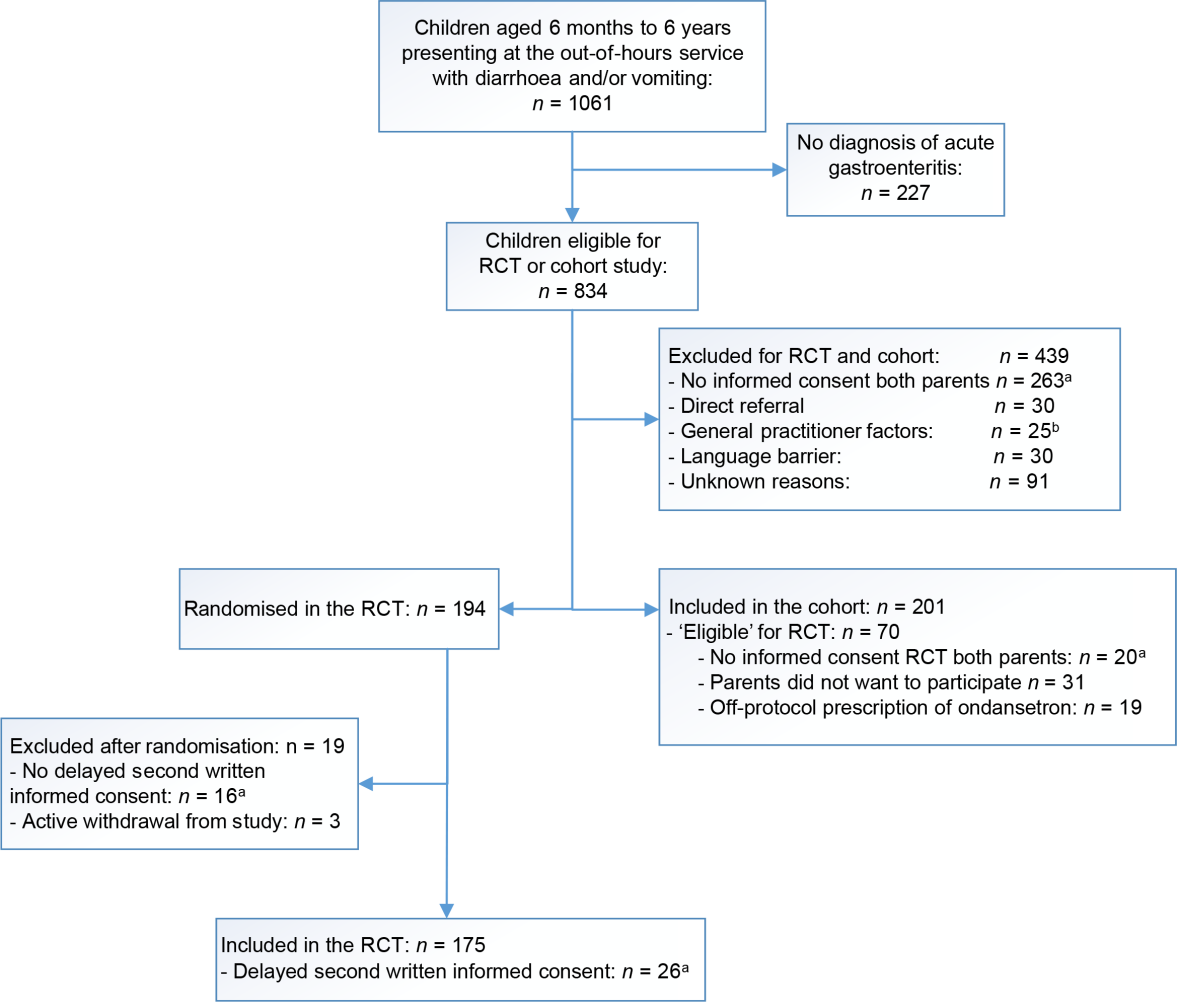
Study flow diagram. RCT = randomised controlled trial. ^a^Only one parent was present (*n* = 325). ^b^GP objected to ondansetron use (*n* = 16) or did not agree with inclusion (*n* = 9)

The eligibility of 1061 children meeting the enrolment criteria were assessed. Of these, 834 had a GP diagnosis of AGE, with 194 ultimately randomised in the RCT and 201 included in the parallel cohort study. In total, 70 children were eligible for both the RCT and parallel cohort study and were included in the study analysis (Figure 2).

### Informed consent

In 39.0% (*n* = 325/834) of cases, only one parent accompanied the child with AGE. Adapting the consent procedure increased the inclusion rate from seven to 10 cases per month. Of the 194 randomised children, 42 were accompanied by only one parent (21.2%) and 16 (8.3%; eight from each RCT group) did not send the second written informed consent after giving verbal permission, so were excluded (Figure 2).

### Off-protocol ondansetron prescribing

GPs prescribed ondansetron off-protocol to 34 (4.1%) of the 834 children with AGE, and 19 of these were eligible for participation in the RCT. There were no clinically relevant differences in baseline characteristics compared with children in the RCT, except that GPs estimated the degree of dehydration to be almost twice as high in these 19 cases compared with those in the RCT (38 versus 20; Table 2).

**Table 2.** Baseline characteristics of all children, and those in each analysed subgroup of children

	Valid *n*	All included children(*n* = 175)	Valid *n*	Eligible, but included in parallel cohort study(*n* = 70)	Valid *n*	GP prescribed ondansetron(*n* = 19)
**Demographics**							
Media age, years (IQR)		175	1.50 (0.9–2.1)	69	1.5 (1.0–2.1)	18	2.0 (1.0–3.0)
Females, *n* (%)		175	88 (50.3%)	70	36 (51.4%)	19	10 (52.6%)
**Symptoms**							
Median weight, kg (IQR)		169	11.0 (9.5–14.0)	61	11.0 (9.1–13.5)	16	12.7 (11.0–16.5)
Median vomiting duration, days (IQR)		174	2.0 (1.0–3.0)	70	2.0 (1.0–3.0)	19	3.0 (2.0–4.0)
Median vomiting frequency per 24 hours (IQR)		171	5.0 (4.0–10.0)	66	7.0 (4.0–10.0)	19	8.0 (6.0–10.0)
Diarrhoea present, *n* (%)		174	124 (71.3%)	70	49 (70.0%)	19	13 (68.4%)
Median diarrhoea duration, days^a^ (IQR)		124	2.0 (1.0–3.0)	49	1.0 (0.0–3.3)	13	3.0 (1.0–4.0)
Median diarrhoea frequency per 24 hours^a^ (IQR)		123	3.0 (2.00–5.0)	44	1.0 (0.0–4.0)	13	1.0 (0.0–4.0)
**Management**							
Median GP-assessed degree of dehydration (0–100), (IQR)		170	20.0 (10.0–40.0)	64	25.0 (10.0–39.0)	16	38.0 (30.0–58.0)
Use of concomitant medication		175	65 (37.1%)	66	19 (28.8%)	19	10 (52.6%)
**Risk factors and alarm symptoms**							
Dehydration: risk factors^b^	1	175	63 (36.0%)	70	22 (31.4%)	19	7 (36.8%)
	≥2	175	**18** (**10.3%**)	70	**2** (**2.9%**)	19	0 (0.0%)
Dehydration: alarm symptoms^c^	1	175	32 (18.3%)	70	5 (7.1%)	19	0 (0.0%)
	≥2	175	2 (1.1%)	70	3 (4.3%)	19	0 (0.0%)

Bold results indicate a significant difference. ^a^Numbers only presented for those participants with diarrhoea.^b^Risk factors assessed at baseline were as follows: ≥6 watery stools or diarrhoea, fever (>38°C), and reduced intake in the last 12 hours.^c^Alarm symptoms assessed at baseline were as follows: confused or decreased consciousness, bradycardia, weak peripheral heartbeat pulsations, capillary refill >4 seconds, skin pitch >4 seconds, cold or marbled extremities, and reduced urine output in the last 24 hours.

### Selection bias

Compared with children in the RCT, the baseline characteristics of the 70 RCT-eligible children in the parallel cohort study did not differ with statistical significance except for the risk factors of dehydration. Children in the parallel cohort study had less risk factors for dehydration compared to children in the RCT (odds ratio = 0.22; 95% confidence interval = 0.11 to 1.00), but the median GP-assessed dehydration level did not differ with statistical significance (*P* = 0.302) (Table 2).

## Discussion

### Summary

Almost 40% of all children attended with one parent, making it difficult to obtain informed consent as required and, thereby, complicating inclusion. GPs also prescribed off-protocol ondansetron if they suspected more severe dehydration, but this did not correspond with known dehydration risk factors or alarm symptoms. Children in the parallel cohort study had fewer additional risk factors for dehydration compared with children in the RCT.

### Strengths and limitations

The authors are aware of no prior research assessing the pitfalls of the trial recruitment process in an OOH-PC. Consecutive children were screened presenting to one of the three OOH-PC over a period spanning more than 2 years, making this study highly representative of the population. Lessons have been learnt from the pilot, and a parallel cohort study has been added in which the children included have been evaluated. However, a limitation is that the RCT-eligible children in the parallel cohort study — to whom GPs prescribed ondansetron — was small (*n* = 19), precluding statistical testing with the children in the RCT. It should also be noted that the recommendations are based on the data from one RCT and a parallel cohort study.

### Comparison with existing literature

#### Informed consent

Based on guidance for research involving humans, the METc decided that both parents must sign a parental consent form.^12^ However, the risk for children in this RCT was deemed low to moderate, with sufficient evidence of effect in referred children and extremely low risk of adverse events.^13–15^ In this study, almost 40% of children diagnosed with AGE at the OOH-PC were accompanied by only one parent, but the acuteness of the clinical problem meant that inclusion and randomisation could not be delayed to obtain the second consent, potentially resulting in study exclusion. This problem with obtaining informed consent from both parents has been reported in other paediatric RCTs.^16,17^

Based on the results of a pilot study that confirmed the above, the METc agreed to an adapted procedure that increased the inclusion rate by three children per month. The adaptation allowed for one parent to give written informed consent and the second to give initial oral informed consent by telephone, with confirmation of written informed consent obtained by post. A second written informed consent for 16 children was not received despite calling repeatedly; but, these children were randomised, eight received the study medication, and some even returned their diary. Nevertheless, they were excluded for protocol deviation, raising ethical concerns given that they had been randomised, had completed study activities, and had received the study medication.

#### Off-protocol ondansetron prescribing

Implementation of the study protocol created more awareness of the potential efficacy of ondansetron for children with AGE. In 34 children, GPs prescribed ondansetron off-protocol despite not being recommended in national guidelines.^9^ After the pilot study, fearing that the effect of ondansetron would be diluted if prescribed in both study arms, it was decided to not include eligible children from the RCT if a GP prescribed ondansetron before randomisation. Their follow-up was monitored in the parallel cohort study instead.

A clinically relevant difference existed in the level of dehydration estimated by the GP between groups, but owing to the small group size (*n* = 19), this was without statistical significance. Among children receiving off-protocol ondansetron, the GP estimated dehydration to be almost twice that in children in the RCT (38 versus 20). Given that it was intended to assess the effect of ondansetron in children at increased risk of dehydration, excluding these may have resulted in an underestimation of the true effect of ondansetron. However, the study demonstrated no differences in risk factors or alarm symptoms of dehydration. Therefore, the level of dehydration estimated by the GP alone should be interpreted with caution.

#### Selection bias

The baseline characteristics of RCT-eligible children in the parallel cohort study did not differ from those of children in the RCT, except that those in the parallel cohort study had fewer risk factors for dehydration. This could imply that the parents of more severely dehydrated children were more willing to participate in the RCT. However, the median dehydration level assessed by the GP did not differ statistically between groups. Other studies have shown that there is no structured way of assessing dehydration in children with AGE,^18,19^ meaning that determining the course of AGE and the risk of dehydration remain important challenges. Further research is needed to evaluate the prognostic value of risk factors and GP-based assessments of dehydration. Given the number of additional risk factors and the GP estimates, the authors are confident that they included children at mild-to-moderate risk of dehydration, as intended.

### Implications for practice

The authors' experiences indicate that there are four areas in which study designs like theirs need improving. First, there is a need to reconsider if written informed consent is required of both parents in pragmatic RCTs involving low risk to the child, especially in acute settings. There is need for future observational research to see how often both parents visit the OOH-PC with their child, as often the other parent stays at home for other caring responsibilities. By identifying the exact numbers for multiple childhood diseases the need to reconsider this ethical decision increases even more. Consistent with this study's approach, it is thought that obtaining the written informed consent of one parent and the oral consent of the second parent, in the presence of the first parent and an RA, is ethically more responsible than excluding a child who otherwise engages in the study but for whom a second written consent form is not received. Second, GPs should receive information that starting with the intervention (or on a paediatrician’s recommendation) during an RCT can seriously bias the outcomes. Communication with all stakeholders, to assess the barriers to protocol compliance, should be routine when performing (pragmatic) RCTs. Third, the authors recommend initiating a parallel cohort study to run alongside RCTs to ensure follow-up when parents do not want to participate in the RCT. This allows comparison of the characteristics of children who did and did not participate, and gives an opportunity to assess if the right population was included. Finally, initiating a pilot study before an RCT offers an invaluable opportunity to evaluate potential barriers to study inclusion and patient selection.

## Trial registration number

Dutch Trial Register number*:* NL5830 (https://www.trialregister.nl/trial/5830)
